# Expression of Estrogen Receptors in Main Immune Organs in Sheep During Early Pregnancy

**DOI:** 10.3390/ijms26083528

**Published:** 2025-04-09

**Authors:** Zhen Yang, Yaqi Zhang, Zhihong Cao, Zhouyuan Li, Leying Zhang, Ling Yang

**Affiliations:** School of Life Sciences and Food Engineering, Hebei University of Engineering, Handan 056038, China; 15031789468@163.com (Z.Y.); 18632804307@163.com (Y.Z.); c48990776@163.com (Z.C.); 13213533399@163.com (Z.L.); zhangly056000@126.com (L.Z.)

**Keywords:** estrogen receptor, immune organ, pregnancy, sheep

## Abstract

Estrogen exerts its action via estrogen receptors (ERs), including ERα and ERβ, and has effects on immunomodulation during pregnancy. It is known that there are changes in the function of the maternal immune organs during pregnancy. However, it is not clear if early pregnancy has effects on the expression of ERα and ERβ in the ovine maternal thymus, lymph nodes, spleen, and liver. In this study, these maternal immune organs were harvested at day 16 of the estrous cycle and at days 13, 16, and 25 of pregnancy (n = 6 for each group) after the ewes were euthanized. The mRNA and protein expression of ERα and ERβ were analyzed using real-time PCR and Western blot and immunohistochemical analyses. The results reveal that the mRNA and protein expression of both ERα and ERβ were upregulated in the maternal spleen and the expression of ERα and ERβ in the thymus, lymph nodes, and liver was modulated during early pregnancy. In conclusion, early pregnancy modulates the expression of ERα and ERβ in the maternal thymus, lymph nodes, spleen, and liver in a tissue-specific manner, which is related to the regulation of maternal immune function during early pregnancy in ewes.

## 1. Introduction

Estrogen not only plays an essential role in the luteinizing hormone peak during the periconceptional period, but also is involved in placentation through the remodeling of the function of immune cells and the expression of angiogenic factors in the uterus during early pregnancy [1]. Estrogen mediates the process of implantation through regulating the expression of estrogen-modulated paracrine factors in the uterus, and these factors can promote cell growth or amplify estrogenic effects during early pregnancy in mice [2]. Estrogen can induce vasodilator production via estrogen receptors (ERs), including ERα and ERβ, in the uterine artery, which is necessary in providing nutrient support for fetal growth and survival during pregnancy in humans [3]. The expression of ERα and ERβ in the endometrium is necessary for normal pregnancy, and ERα is related to uterine cell proliferation, but ERβ has a negative effect on ERα action [4]. A high level of estrogen enhances the proportions of CD4^+^ CD25^+^ Treg in peripheral blood, which is essential for estrogen-mediated immunomodulation during pregnancy in humans [5]. In addition, early pregnancy modulates uterine *ER* gene expression in a tissue- and cell type-specific manner in sheep [6].

There is a dynamic adaptation in the maternal immune system during pregnancy, which contributes to a tolerance toward the allogeneic fetus, but not a broad maternal immune inhibition [7]. Pregnant women infected with COVID-19 or Zika virus show changes in maternal cell immunity that increases the risk of preeclampsia and preterm birth [8,9]. Hormone replacement therapy with estrogen–androgen is found to improve reproductive performance [10]. Estrogens secreted from the ovary and placenta can modulate immune system development and immune responses via their receptors expressed in the immune cells, which is involved in the adaptation of the organism to the intrinsic physiological changes in females [11]. Estrogen can suppress autophagy of the immune system via ERα and ERβ, which plays a key role in pre-implantation and maintaining embryo survival [12]. In addition, estrogens have effects on innate and adaptive immunity, which modulate the activity of different immune cells in the thymus, spleen, and lymph nodes through ERα and ERβ [13].

**Figure 1:**
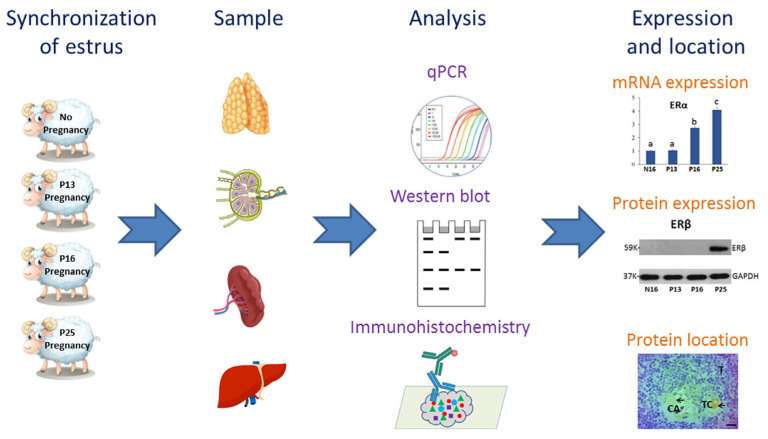
Overview of the experimental approach.

In ruminants, conceptus signal (interferon-tau, IFNT) and high levels of progesterone modulate the maternal innate immune system [14], which changes immune function both locally and systemically during pregnancy. IFNT has paracrine and endocrine actions in modulating maternal innate immune functions, which is essential for avoiding conceptus rejection by the mother during early pregnancy in ruminants [15]. Our previous studies report that IFNT has effects on bone marrow, thymus, spleen, lymph nodes, and liver through an endocrine manner around day 16 of pregnancy in ewes. In sheep, early pregnancy has effects on the expression of melatonin receptors, gonadotropin-releasing hormone and its receptor, and prolactin and its receptor in the ovine thymus, lymph nodes, spleen, and liver [16], which modulates the function of the maternal immune system via endocrine and/or paracrine/autocrine pathways in a tissue-specific manner. However, it is not clear if early pregnancy has effects on the expression of ERα and ERβ in the ovine maternal thymus, lymph nodes, spleen, and liver, which may be involved in the modulation of maternal immune function. Humans and ruminants belong to mammals and may be regulated in the same way. The aim of the present study was to investigate the expression of ERα and ERβ in these organs from nonpregnant ewes and early pregnant ewes (Figure 1), which will contribute to understanding pregnancy immune tolerance. In addition, estrogen or estrogen inhibitors may be used to improve maternal immune tolerance and decrease embryo loss.

## 2. Results

### 2.1. ERα and ERβ in the Thymus

**Figure 2:**
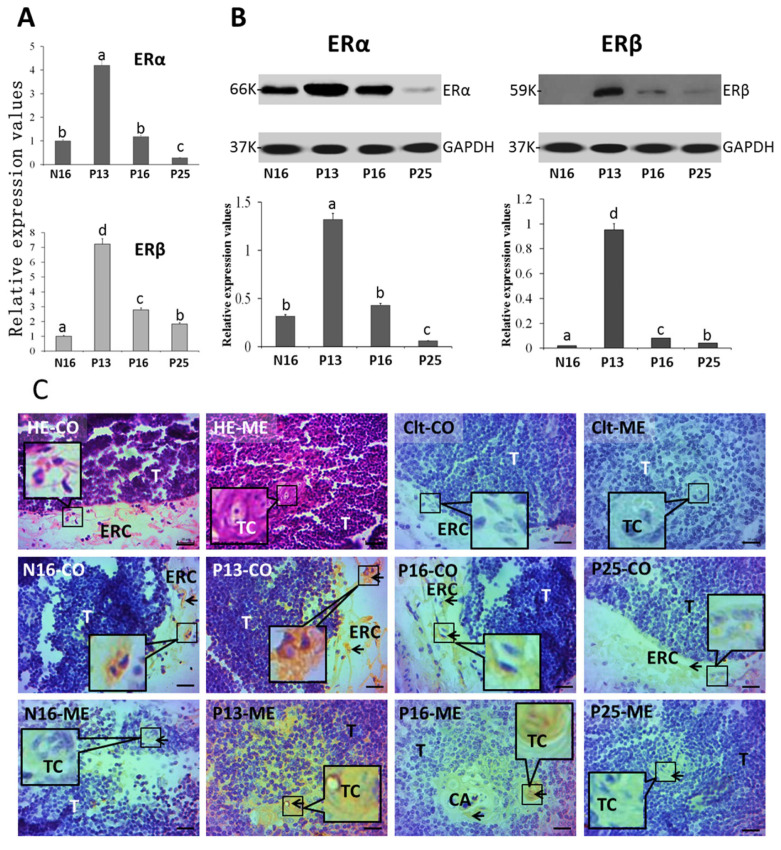
Expression of mRNAs (**A**) and proteins (**B**) of ERα and ERβ and immunohistochemical localization of the ERα protein (**C**) in the thymus. Note: CO = cortex; ME = medulla; Clt = negative control; T = thymocyte; ERC = epithelial reticular cell; CA = capillary; TC = thymic corpuscle; Bar = 20 µm. Positive signals are indicated by arrows. Different letters (a, b, c, and d) within columns indicate significant differences (*p* < 0.05).

Figure 2A indicates that the expression values of *ERα* mRNA (N16, 1; P13, 4.22 ± 0.36; P16, 1.18 ± 0.08; P25, 0.28 ± 0.03) and protein (N16, 0.32 ± 0.02; P13, 1.32 ± 0.11; P16, 0.43 ± 0.03; P25, 0.06 ± 0.01) were the highest at P13 (*p* < 0.05) in the maternal thymus but were the lowest at P25 (*p* < 0.05) among the four groups. Furthermore, the expression values of *ERβ* mRNA (N16, 1; P13, 7.23 ± 0.57; P16, 2.78 ± 0.19; P25, 1.83 ± 0.12) and protein (N16, 0.02 ± 0.00; P13, 0.95 ± 0.07; P16, 0.08 ± 0.01; P25, 0.04 ± 0.01) were also the highest at P13 among the four groups (*p* < 0.05) but were the lowest at N16 among the four groups (*p* < 0.05). ERβ protein was almost undetected at N16 and P25 and weakly expressed in P16 compared to P13 (*p* < 0.05). In addition, the ERα protein was located in the epithelial reticular cells, capillaries, and thymic corpuscles. The staining intensities for ERα protein were 1, 3, 2, and 0.5 for N16, P13, P16, and P25, respectively (Figure 2C), which were almost consistent with the protein expression of ERα. The staining intensity was as follows: 0 = negative; 1 = weak; 2 = moderate; 3 = stronger.

### 2.2. ERα and ERβ in Lymph Nodes

**Figure 3:**
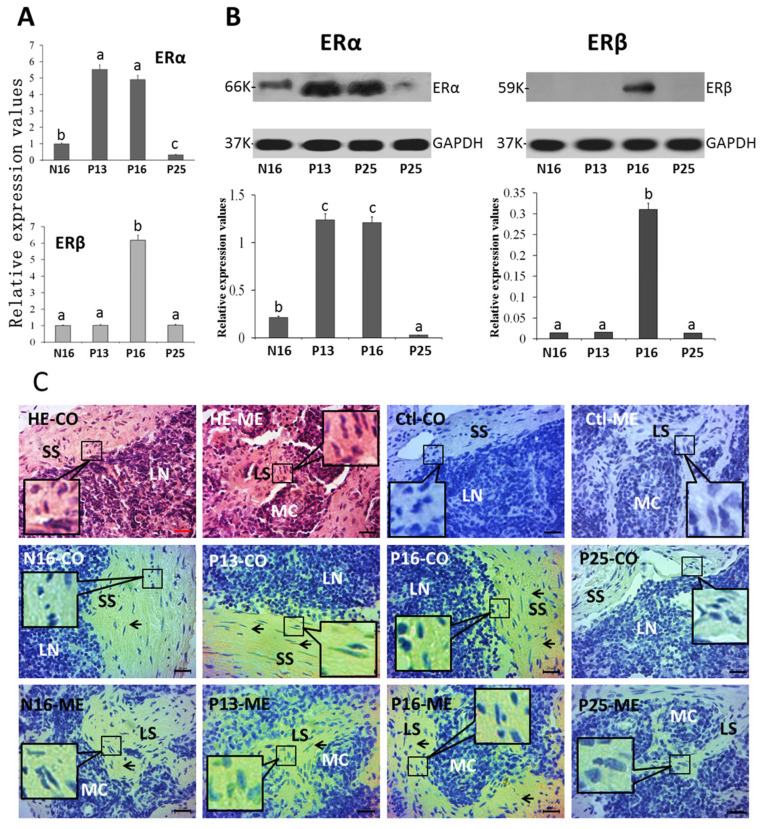
Expression of mRNAs (**A**) and proteins (**B**) of ERα and ERβ and immunohistochemical localization of the ERα protein (**C**) in the lymph nodes. Note: CO = cortex; ME = medulla; SS = subcapsular sinus; TR = trabeculae; LN = lymphoid nodule; LS = lymph sinus; MC = medullary cord; Bar = 20 µm. Positive signals are indicated by arrows. Different letters (a, b, and c) within columns indicate significant differences (*p* < 0.05).

*ERα* mRNA (N16, 1; P13, 5.52 ± 0.43; P16, 4.91 ± 0.37; P25, 0.33 ± 0.02) and protein (N16, 0.22 ± 0.02; P13, 1.24 ± 0.85; P16, 1.21 ± 0.72; P25, 0.03 ± 0.00) values were higher at P13 and P16 than at N16 (*p* < 0.05) and P25 (*p* < 0.05; Figure 3A), and the *ERα* mRNA and protein values were the lowest at P25 among the four groups (*p* < 0.05). In addition, *ERβ* mRNA (N16, 1; P13, 1.02 ± 0.08; P16, 6.18 ± 0.48; P25, 1.08 ± 0.09) and protein (N16, 0.02 ± 0.00; P13, 0.02 ± 0.00; P16, 0.31 ± 0.02; P25, 0.02 ± 0.00) peaked at P16 (*p* < 0.05) and ERβ protein was almost undetected at N16, P13, and P25. The ERα protein was located in the subcapsular sinus and lymph sinus, and the staining intensities for ERα protein were 1, 3, 3, and 0 for N16, P13, P16, and P25, respectively (Figure 3C), which were almost consistent with the protein expression of ERα.

### 2.3. ERα and ERβ in the Spleen

**Figure 4:**
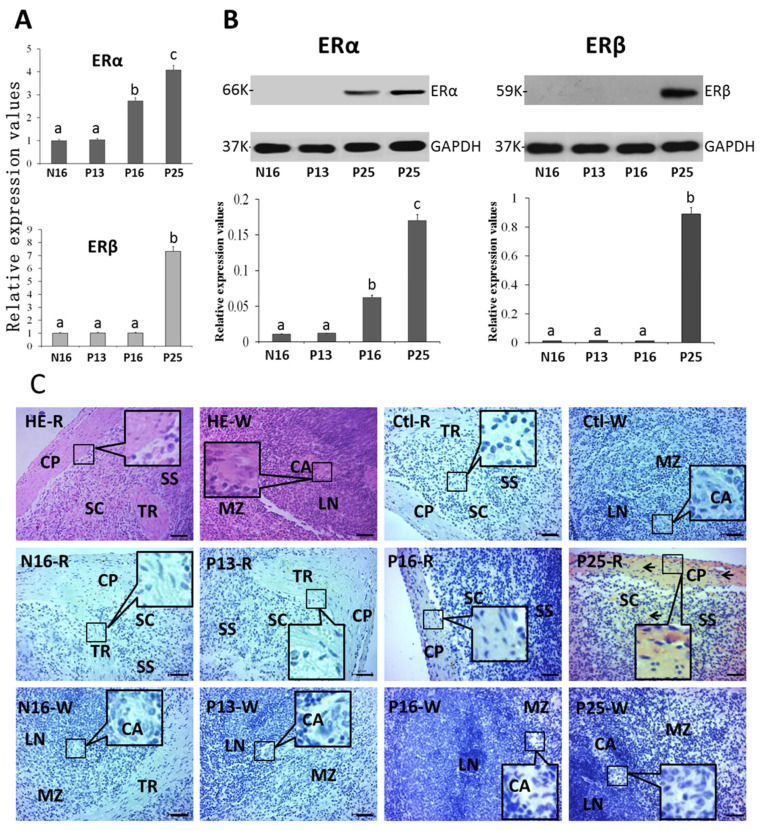
Expression of mRNAs (**A**) and proteins (**B**) of ERα and ERβ and immunohistochemical localization of the ERα protein (**C**) in the spleen. Note: R = red pulp; W = white pulp; CP = capsule; TR = trabeculae; SS = splenic sinus; SC = splenic cord; MZ = marginal zone; LN = lymphoid nodule; CA = central arteriole; Bar = 50 µm. Positive signals are indicated by arrows. Different letters (a, b, and c) within columns indicate significant differences (*p* < 0.05).

There were increases in the expression of *ERα* mRNA (N16, 1; P13, 1.04 ± 0.07; P16, 2.74 ± 0.16; P25, 4.08 ± 0.31) and protein (N16, 0.01 ± 0.00; P13, 0.01 ± 0.00; P16, 0.06 ± 0.01; P25, 0.17 ± 0.01) at P16 and P25 compared to N16 (*p* < 0.05) and P13 (Figure 4A; *p* < 0.05) in the spleen, and ERα protein was almost undetected at N16 and P13. *ERβ* mRNA (N16, 1; P13, 1.01 ± 0.06; P16, 1.02 ± 0.07; P25, 7.32 ± 0.53) and protein (N16, 0.01 ± 0.00; P13, 0.01 ± 0.00; P16, 0.01 ± 0.00; P25, 0.89 ± 0.38) peaked at P25 (*p* < 0.05), and ERβ protein was almost undetected at N16, P13, and P16. In addition, ERα protein was located in the capsule, trabeculae, and splenic cords and the staining intensities for ERα protein were 0, 0, 1, and 2 for N16, P13, P16, and P25, respectively (Figure 4C), which were almost consistent with the protein expression of ERα.

### 2.4. ERα and ERβ in the Liver

**Figure 5:**
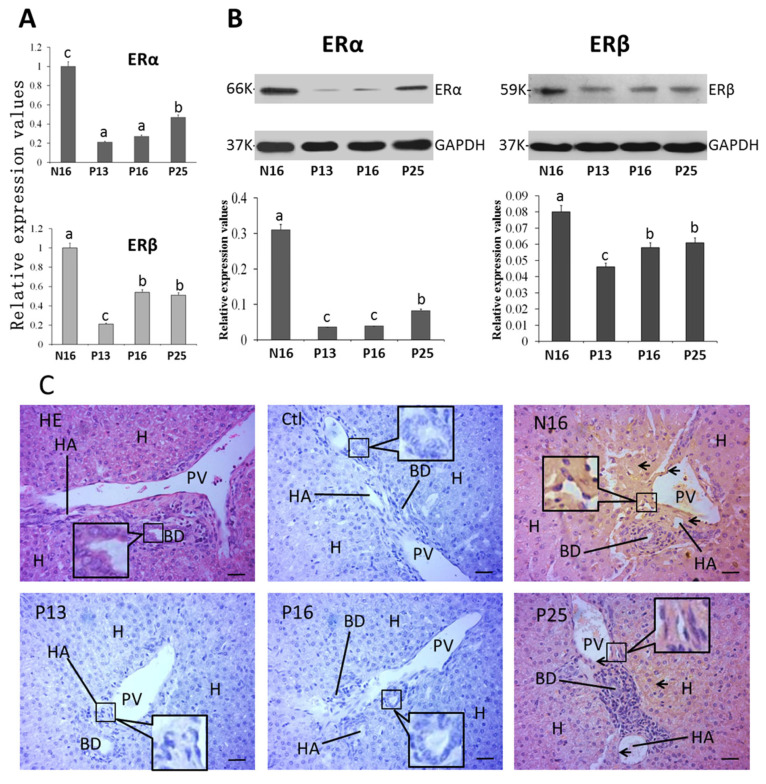
Expression of mRNAs (**A**) and proteins (**B**) of ERα and ERβ and immunohistochemical localization of the ERα protein (**C**) in the liver. Note: HA = hepatic artery; PV = portal vein; BD = bile ductile; H = hepatocyte; Bar = 50 µm. Positive signals are indicated by arrows. Different letters (a, b, and c) within columns indicate significant differences (*p* < 0.05).

The mRNA (N16, 1; P13, 0.21 ± 0.01; P16, 0.27 ± 0.02; P25, 0.47 ± 0.03) and protein (N16, 0.31 ± 0.02; P13, 0.04 ± 0.00; P16, 0.04 ± 0.00; P25, 0.08 ± 0.01) levels of ERα were decreased at P13 and P16 compared with N16 (*p* < 0.05) and P25 (*p* < 0.05), and the level of ERα at N16 was the highest among the four groups (*p* < 0.05). In addition, the expression of *ERβ* mRNA (N16, 1; P13, 0.21 ± 0.02; P16, 0.54 ± 0.03; P25, 0.51 ± 0.03) and protein (N16, 0.08 ± 0.01; P13, 0.04 ± 0.00; P16, 0.06 ± 0.01; P25, 0.06 ± 0.01) was inhibited during early gestation (*p* < 0.05), but there was an increase in the expression of *ERβ* mRNA and protein at P16 and P25 compared to P13 (Figure 5A,B). ERα protein was located in the hepatocyte, endothelial cells of the proper hepatic arteries, and hepatic portal veins (Figure 5C), and the staining intensities for ERα protein were 3, 0, 0, and 1 for N16, P13, P16, and P25, respectively, which were almost consistent with the protein expression of ERα.

## 3. Discussion

The thymus is responsible for the generation of a T-cell receptor repertoire, which responds to foreign antigens, provides surveillance, and remains tolerant to self [17]. Pregnancy induces a severe reduction in thymus size and thymocyte output, which is associated with the suppression of chemokines in the thymic nonlymphoid cells during pregnancy [18]. Our previous studies report that the immune signaling pathways, including complement pathway, nod-like receptor pathway, and toll-like receptor pathway, as well as kappa B subunits and IkappaB protein, are changed in the maternal thymus [19], which is involved in the maternal immune regulation in sheep. Estrogens regulate the expression of autoimmune regulator (AIRE) via ERs, which modulates the expression of tissue-restricted antigen genes to generate self-peptides to bind to major histocompatibility complex molecules in the medullary thymic epithelial cells (TECs) [20]. Estrogen can also modulate the development and differentiation of T cells and the immune functions of the TECs, which are involved in autoimmunity via ERs [21].

During the proestrus phase in the mouse thymus, a high level of estradiol decreases the percentage of CD4^+^CD8^+^ double-positive T-cells via ERα, but in the proestrus phase, CD4^+^CD8^−^ or CD4^−^CD8^+^ single-positive T-cells are significantly enhanced [22]. In addition, during the first half of pregnancy, estriol is implicated in regulating the processes of myeloid dendritic cell maturation in the thymus and is also related to the steroid-induced involution of the thymus [23]. There is an upregulation of estrogen levels during pregnancy, which suppresses thymocyte proliferation and leads to thymic involution during pregnancy in mice [24]. Our data reveal that mRNA and protein levels of ERα and ERβ upregulated at P13, but there was a downregulation of ERα and ERβ levels at P25. Thus, it is supposed that estrogens have effects on thymic function via ERα and ERβ in an endocrine manner during early pregnancy in sheep.

The lymph nodes consist of antigen-presenting cells and antigen-responsive cells, which are involved in the recruitment of naïve lymphocytes and antigen-presenting cells, the generation of adaptive immune responses, and the suppression of autoreactive cells [25]. The presence of an embryo changes the function of the lymph nodes around the reproductive tract, which is implicated in regulating immune responses in the maternal local immune tissues during early pregnancy in the ovine [26]. It has been reported that prostaglandin synthases, T helper cell cytokines, nod-like receptors, toll-like receptors, complement components, as well as nuclear factor kappa B family, are modulated in ovine lymph nodes [27], which is related to the maternal immune regulation in ewes. ERα is expressed by the follicular dendritic cells in lymph nodes, which is related to breast cancer and inflammation in humans [28]. Our data manifest that the expression level of ERα peaked at P13 and P16 and the level of ERβ peaked only at P16. However, the expression levels of ERα and ERβ downregulated at P25. Therefore, ERα and ERβ are involved in the immunoregulation of maternal lymph nodes during early pregnancy, which is in an endocrine manner in ewes.

The spleen comprises red pulp (RP) and white pulp (WP), and the WP is the primary immunologic region, and the (RP) has an immune function distinct from the WP. The marginal zone (MZ) is between the WP and RP, and there are many macrophages in the MZ [29]. Estrogen has effects on the expression of some cytokines in the spleen dendritic cells from the female mice, which is through ERα [30]. 17β-estradiol can regulate the function of mice spleen B cells, which is via toll-like receptor 9 [31]. In addition, 17β-estradiol has beneficial effects on the function of splenic CD4^+^ T lymphocytes, which is mediated by ERα, but not ERβ, and related to the suppression of endoplasmic reticulum stress [32]. Early pregnancy increases the level of ERα and the percentage of mature macrophages in the spleen of female mice, which plays a cardinal role in regulating immune phenomena during pregnancy [33]. It has been reported that early pregnancy has effects on the expression of T helper cell cytokines, toll-like receptors, and nuclear factor kappa B family in ovine spleen [27,34], which is implicated in maternal immune adaptation. Our results reveal that early pregnancy enhanced the expression of ERα and ERβ. Therefore, the upregulation of ERα and ERβ is implicated in modulating maternal splenic function during early pregnancy through an endocrine manner.

As the largest gland of the body, the liver plays cardinal roles in immunologic responses and nutrient metabolism and there are necessary hepatic adaptations in immunology and nutrition during normal pregnancy [35]. The *ERα* mRNA level in the liver is enhanced from days 7 to 21 of pregnancy, and the expression level of *ERβ* is variable during pregnancy, which is related to hepatic synthetic functions in rats [36]. Estrogen can change the cytokine/chemokine repertoire in the liver via ERs, which are involved in modulating the liver immunosuppressive microenvironment [37]. 17β-estradiol supplementation can improve mitochondrial function to prevent nonalcoholic fatty liver disease via the ERα pathway in bilateral oophorectomy female rats [38]. Previous reports show that early pregnancy modulates the expression of Th cytokines, toll-like receptor pathway, nuclear factor kappa B signaling, complement components, nod-like receptors, and CD4, which is associated with the hepatic local immunosuppressive milieu in sheep [35]. Our results show that early pregnancy suppressed the expression of ERα and ERβ, but there were increases in the expression levels of ERα and ERβ from P13 to P25. Therefore, the changes in the expression of hepatic ERα and ERβ are involved in regulating maternal hepatic function, including the immune function, during early pregnancy.

**Figure 6:**
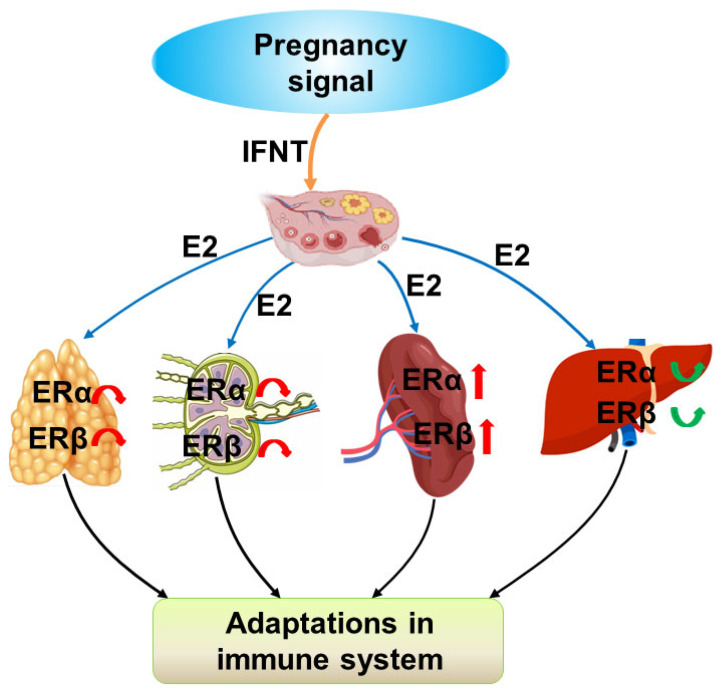
Sketch of expression of estrogen receptors (ERs) in the ovine thymus, lymph nodes, spleen, and liver during early pregnancy. Early pregnancy signal (IFNT) exerts effects on the ovary, which modulates the secretion of estradiol (E2); leads to changes in the expression of ERα and ERβ in the maternal thymus, lymph nodes, spleen, and liver; and contributes to the adaptations in the maternal immune system.

It has been reported that pregnancy has effects on estrogen levels [24], which may modulate the expression of ERα and ERβ in the maternal thymus, lymph nodes, spleen, and liver via an endocrine manner. Therefore, estrogen exerts its actions on these maternal immune organs via ERα and ERβ in a tissue-specific manner to regulate maternal immune function during early pregnancy (Figure 6). Furthermore, estrogen or estrogen inhibitors may be used to modulate maternal immune tolerance and improve fertility, and, thus, herd profitability.

## 4. Materials and Methods

### 4.1. Animals and Experimental Design

The experiments were performed as described previously [16]. Ewes (Small-tail Han) approximately 18 months of age were housed at local farm of Handan in China. Twenty-four ewes (n = 6 for each group) were checked twice daily after synchronization of estrus, and animals that manifested a mating mark (day 0) were assigned to collect thymus, lymph nodes, spleen, and liver on days 13, 16, and 25 of gestation (P13, P16, and P25) after natural mating and on day 16 of the estrous cycle (N16). These four different phases were selected based on secretion of progesterone and IFNT as described in a previous report [12]. Pregnancy was confirmed by finding a conceptus in the uterus after the ewes were euthanized. Tissue pieces of thymus, lymph nodes, spleen, and liver were collected and fixed with fresh 4% paraformaldehyde and also rapidly frozen at −80 °C until RNA isolation and protein analysis.

### 4.2. RNA Extraction and RT-qPCR Assay

Total RNA was extracted from these samples using TRNzol Universal Reagent (DP424; Tiangen Biotech Co., Ltd., Beijing, China), and RT-qPCR was performed as described previously [16]. Primers (Table 1) of ovine *ERα* and *ERβ* genes were designed and synthesized by Shanghai Sangon Biotech Co., Ltd. (Shanghai, China). The 2^−ΔΔCt^ analysis method was applied to analyze the relative levels [39].

### 4.3. Western Blot Analysis

Western blot analysis was carried out as described previously [12]. A rat anti-ERα monoclonal antibody (Santa Cruz Biotechnology, Santa Cruz, CA, USA, sc-53490) or mouse anti-ERβ antibody (Abcam, Cambridge, UK, ab187291) was employed to analyze the expression of ERα or ERβ protein in these tissues in a 1:1000 dilution. The primary antibodies were validated by specific binding to native ovine proteins. An anti-GAPDH antibody (Santa Cruz Biotechnology, sc-47724) was used for normalizing expression values.

### 4.4. Immunohistochemistry Analysis

Immunohistochemistry was performed as described previously [16], and the ERα antibody (Santa Cruz Biotechnology, sc-53490) was employed at 4 °C overnight in a humidified chamber in a 1:200 dilution. After washing three times, incubation with the secondary antibody (Biosharp, Hefei, China, BL002A) diluted at 1:500 followed using the application of a DAB kit (Tiangen Biotech) as the chromogen. The sections were counterstained using hematoxylin. The negative control was prepared by substituting the primary antibody with an antiserum-specific isotype at the same protein concentration. The images were analyzed as described previously [16].

### 4.5. Statistical Analysis

Proc Mixed models of SAS (Version 9.4; SAS Institute, Cary, NC, USA) were utilized for statistical analysis. The data of *ERα* and *ERβ* mRNA and proteins were in a normal distribution after evaluated for normality of the distribution. Then, ANOVA with a post hoc Tukey test was used. *p* < 0.05 was considered significant.

## 5. Conclusions

It is reported for the first time that early pregnancy has tissue-specific effects on the expression of ERα and ERβ in the maternal thymus, lymph nodes, spleen, and liver, which regulates the function of main immune organs via an endocrine manner. Therefore, it is suggested that early pregnancy modulates the expression of ERα and ERβ in these immune organs, which is related to the adaptations in the maternal immune system via an endocrine manner during early pregnancy in ewes. Further studies may be focued on the effects of estrogen or estrogen inhibitors on maternal immune tolerance and improving fertility.

## Figures and Tables

**Table 1:** Primers used for RT-qPCR.

Gene	Primer	Sequence	Size (bp)	Accession Numbers
*ERα*	Forward	CTGCTGCTGGAGATGCTGGATG	88	XM_042253635.1
	Reverse	GCTGGCTCTGATTCACGTCTTCC		
*ERβ*	Forward	TGCTGCTGGAGATGCTGAATGC	112	NM_001009737.1
	Reverse	GGTTTCTGGGAGCCCTCTTTGC		
*GAPDH*	Forward	GGGTCATCATCTCTGCACCT	176	NM_001190390.1
	Reverse	GGTCATAAGTCCCTCCACGA		

## Data Availability

Data supporting the findings of this study are available within the paper.
